# Supplementary material for: The First Synthesis of Periodic and Alternating Glycopolymers by RAFT Polymerization: A Novel Synthetic Pathway for Glycosaminoglycan Mimics
Source: Polymers (Basel). 2019 Jan 5;11(1):70. doi: 10.3390/polym11010070 (PMC6401991; doi:10.3390/polym11010070)
Supplement: Supplementary file 1 [file polymers-11-00070-s001.pdf]

Supplementary Materials

The first synthesis of periodic and alternating glycopolymers by RAFT polymerization: A novel synthetic pathway for glycosaminoglycan mimics

Masahiko Minoda[1,]* Tomomi Otsubo[1], Yohei Yamamoto[1], Jianxin Zhao [2], Yoshitomo Honda[3], Tomonari Tanaka[4] and Jin Motoyanagi[1]

1 Faculty of Molecular Chemistry and Engineering, Graduate School of Science and Technology, Kyoto Institute of Technology, Matsugasaki, Sakyo-ku, Kyoto 606-8585, Japan; m7618002@edu.kit.ac.jp (T. O.); m5618030@edu.kit.ac.jp (Y. Y.); jinmoto@kit.ac.jp (J. M.)

2 Department of Orthodontics, Osaka Dental University; 8-1, Kuzuhahanazonocho, Hirakata, Osaka, 573-1121, Japan; jianxinzhao@hotmail.com

3 Institute of Dental Research, Osaka Dental University, 8-1, Kuzuhahanazonocho, Hirakata, Osaka, 573-1121, Japan; honda-y@cc.osaka-dent.ac.jp

4 Department of Biobased Materials Science, Graduate School of Science and Technology, Kyoto Institute of Technology, Matsugasaki, Sakyo-ku, Kyoto 606-8585, Japan; t-tanaka@kit.ac.jp

* Correspondence: minoda@kit.ac.jp; Tel.: +81-75-724-7513 (M. M.)

1. ^{1}H and ^{13}C NMR spectra of vinyl monomers (MalVE, LacVE and MalMI)

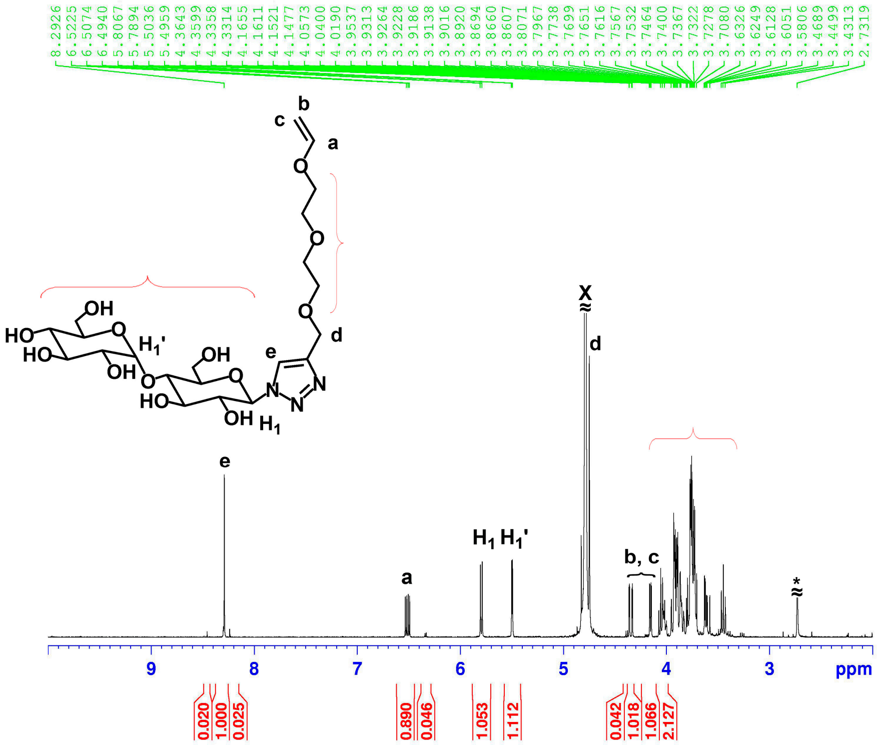

Fig. S1-1. ^{1}H NMR spectrum of **MalVE** in D_2O (x and *; remaining solvents).

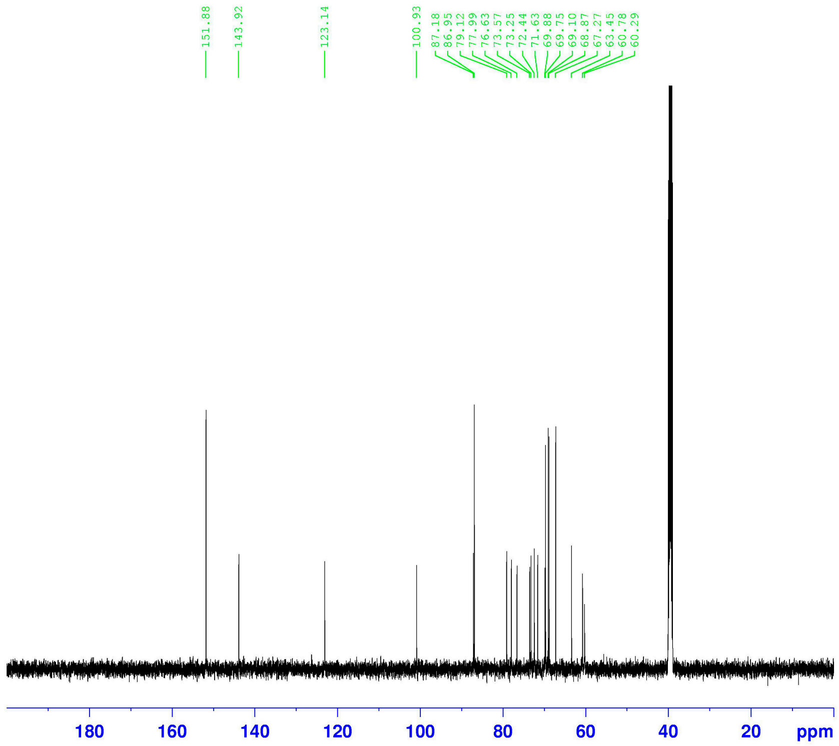

Fig. S1-2. ^{13}C NMR spectrum of **MalVE** in D_2O.

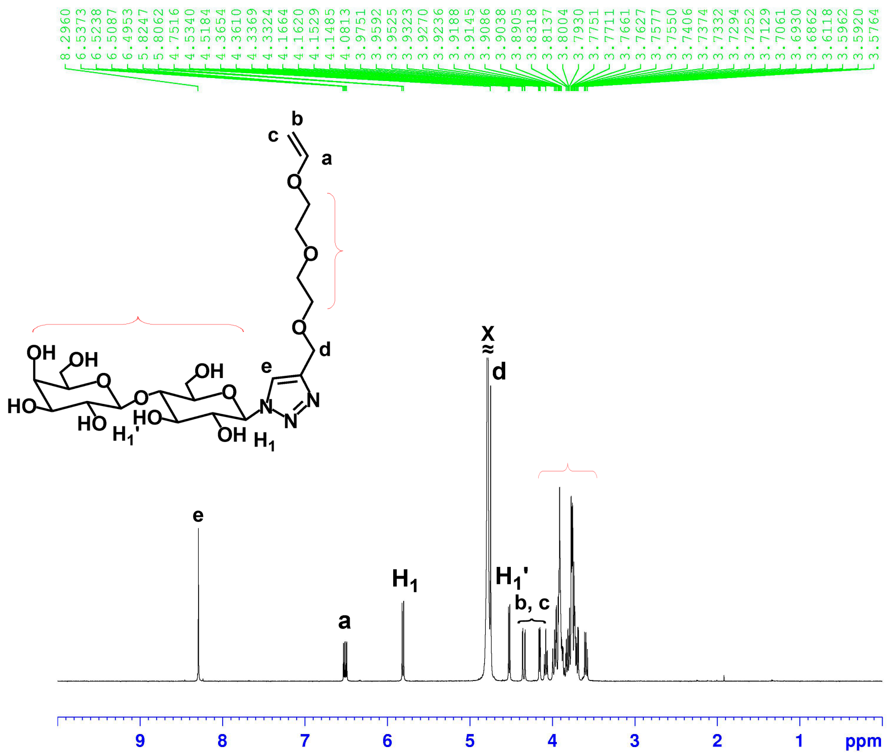

Fig. S1-3. ^{1}H NMR spectrum of **LacVE** in D_2O (x; remaining solvent).

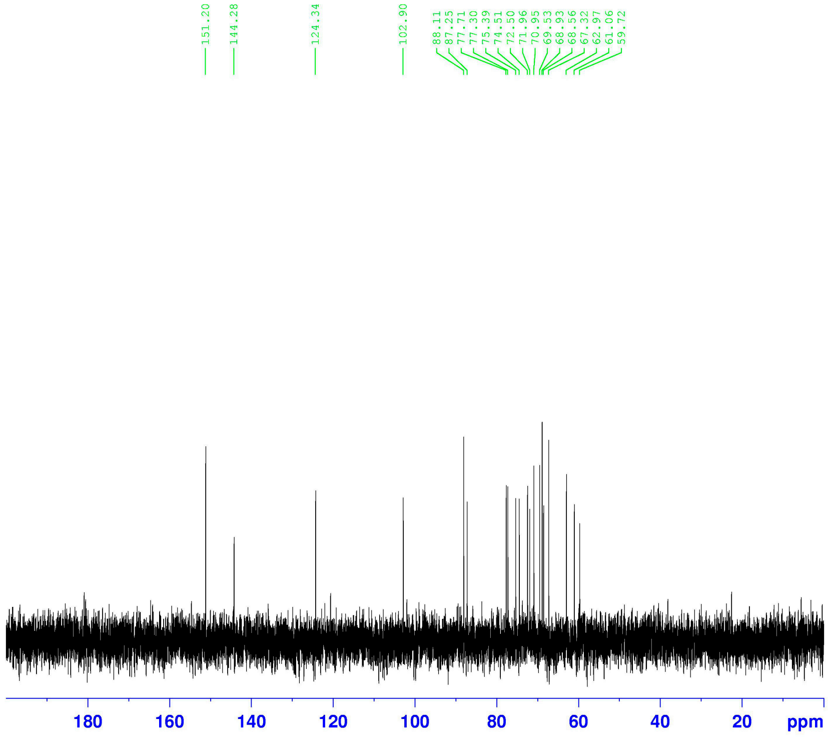

Fig. S1-4. ^{13}C NMR spectrum of **LacVE** in D_2O.

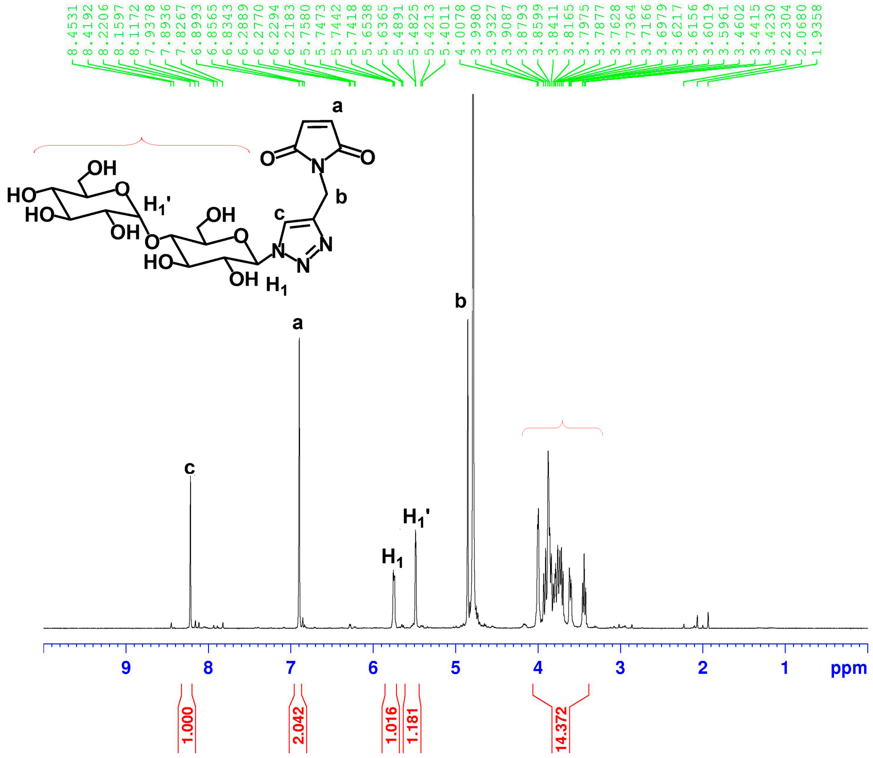

Fig. S1-5. ^{1}H NMR spectrum of **MalMI** in D_2O (x; remaining solvent).

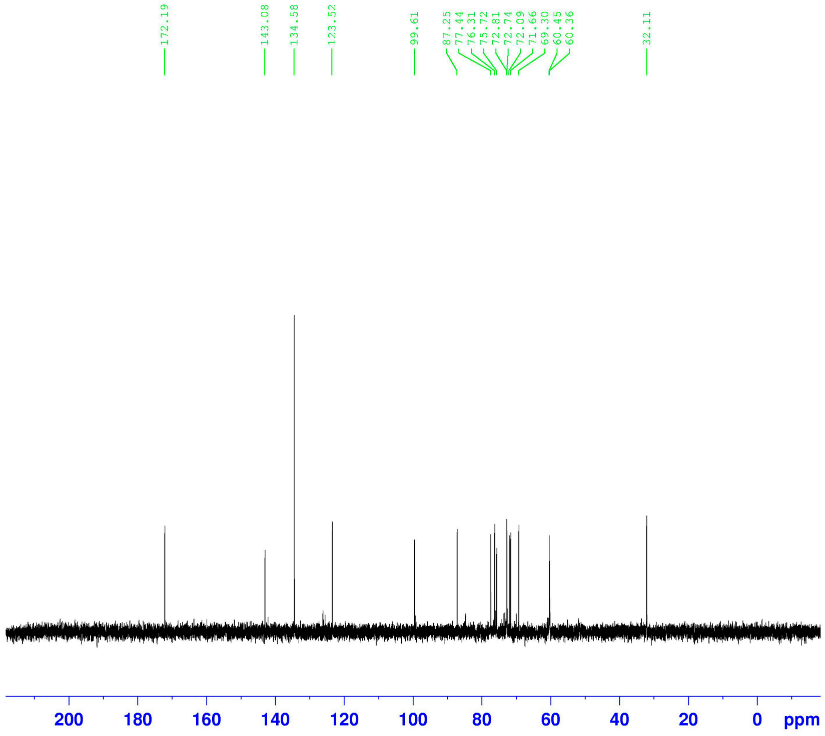

Fig. S1-6. ^{13}C NMR spectrum of **MalMI** in D_2O.

2. Comparison of copolymerization of MalVE and EtMI with and without RAFT agent

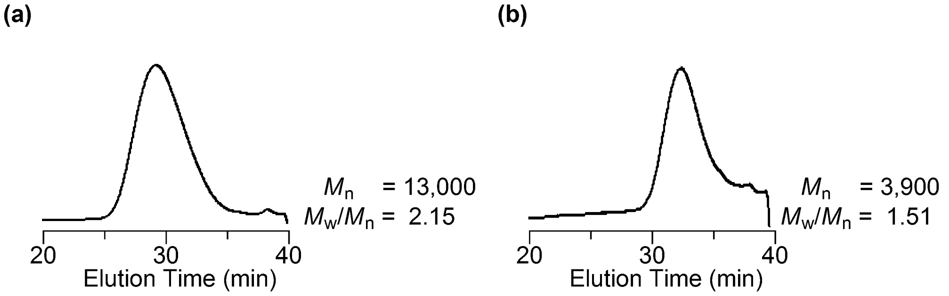

Fig. S2. SEC curves of poly(MalVE-*co*-EtMI) obtained in the radical copolymerization (a) without and (b) with RAFT agent using 0.2 mol L^{-1} $NaNO_3$ aq. as the eluent.

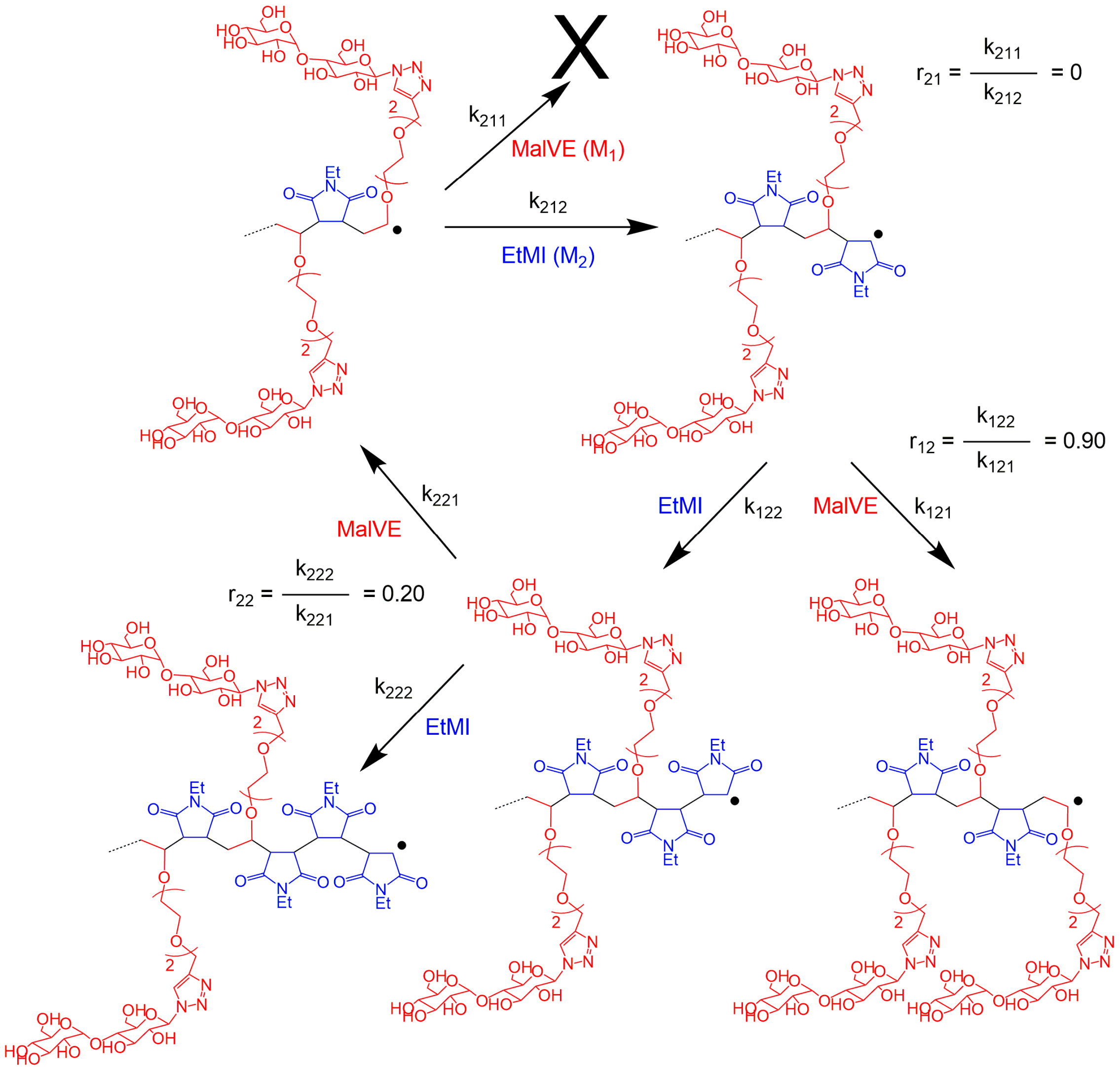

Scheme S1. Schematics of radical copolymerization of MalVE (M_1) and EtMI (M_2).

3. RAFT copolymerization of LacVE and EtMI.

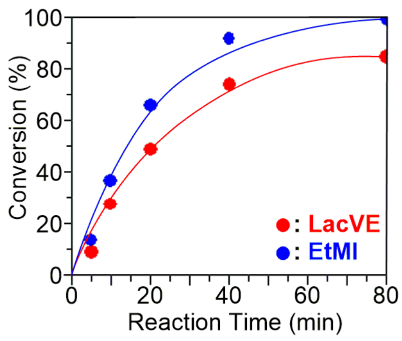

Fig. S3. Time-conversion curves for the RAFT copolymerization of LacVE and EtMI with BTSE.

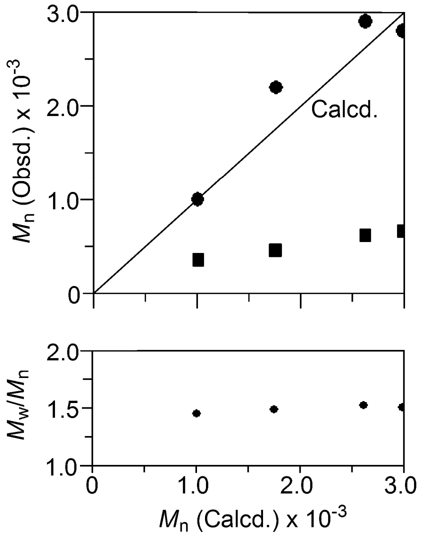

Fig. S4. Experimentally observed M_n and M_w/M_n value of poly(LacVE-*co*-EtMI) plotted against theoretical M_n of poly(LacVE-*co*-EtMI). Filled circles and squares correspond to the M_n data obtained by ^{1}H NMR and SEC, respectively.

4. RAFT copolymerization of LacVE and MalMI.

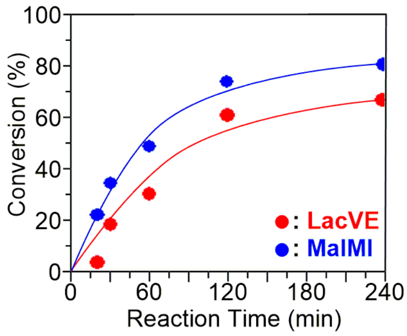

Fig. S5. Time-conversion curves for the RAFT copolymerization of LacVE and MalMI with BTSE.

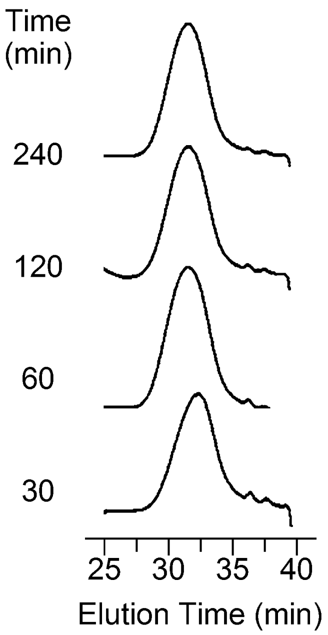

Fig. S6. SEC curves of poly(LacVE-*co*-MalMI) using 0.2 mol L^{-1} $NaNO_3$ aq. as the eluent.

5. Lectin binding assay

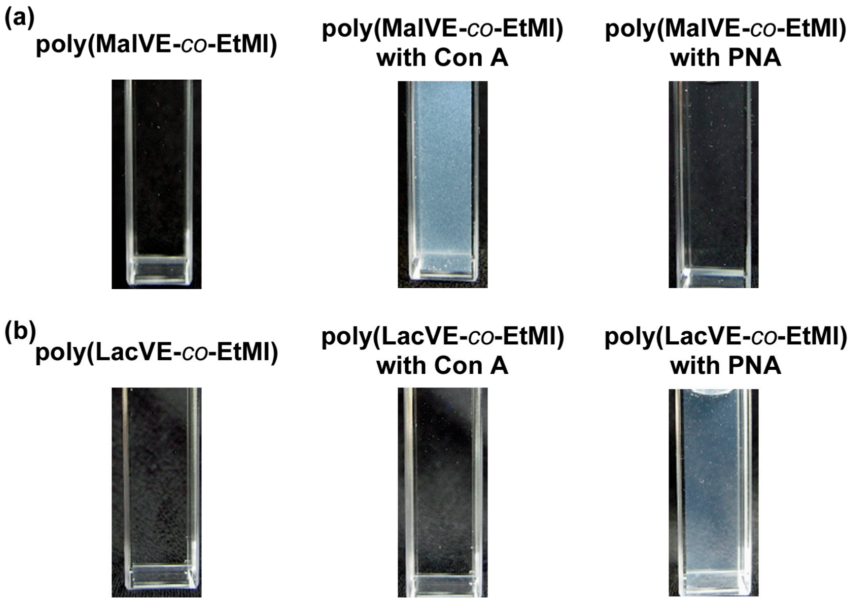

Fig. S7. Photography of (a) poly(MalVE-*co*-EtMI) and (b) poly(LacVE-*co*-EtMI) solution before and after the addition of FITC-unlabeled Con A or PNA.
